# Clinical characteristics associated with primary cardiac angiosarcoma outcomes: a surveillance, epidemiology and end result analysis

**DOI:** 10.1186/s40001-019-0389-2

**Published:** 2019-08-19

**Authors:** Chanqiong Zhang, Chongan Huang, Xueke Zhang, Liang Zhao, Dan Pan

**Affiliations:** 1 Department of Pathology, Wenzhou People’s Hospital, Wenzhou Third Clinical Institutes of Wenzhou Medical University, 299 Guan Rd. Ouhai District, Wenzhou, Zhejiang China; 2 Department of Spine Surgery, Zhejiang Spine Surgery Centre, The Second Affiliated Hospital and Yuying Children’s Hospital of Wenzhou Medical University, Second Medical School of Wenzhou Medical University, Wenzhou, Zhejiang China; 3 Region Information Technology Shanghai Co., Ltd., Jiading District, Shanghai, China

**Keywords:** SEER, Primary cardiac angiosarcoma, Prognostic factors

## Abstract

**Background:**

Primary cardiac angiosarcoma (PCAS) is a rare type of tumour. Furthermore, descriptions of the demographic features and prognostic factors of PCAS patients have been poorly reported.

**Methods:**

A population cohort study was conducted using retrospectively extracted data from the SEER (Surveillance, Epidemiology and End Results) database for patients with histological diagnoses of PCAS; the extracted information included demographic, treatment and outcome data.

**Results:**

A total of 168 cases of PCAS from 1973 to 2013 were included. The mean age at diagnosis was 44.4 ± 15.5 years. PCAS was more prevalent in men than in women. The majority of PCAS patients were white (67.3%), while the incidence of PCAS in black individuals was relatively infrequent (19.0%). In addition, 87 cases were classified as distant stage, 44 as regional stage, and 33 as localized stage. The median disease-specific survival (DSS) was 7.22 months, and the 1-, 2- and 5-year DSS rate for PCAS patients was 34.7%, 14.3% and 10.2%, respectively. Further multivariate analyses showed that an age at (greater than or equal to) 45 years (HR 2.165), no radiotherapy (HR 1.629), tumour size > 5 cm (HR 3.182), and the summary stage was associated with worse PCAS-related survival. Cancer-directed surgery and radiotherapy significantly improved the DSS for patients with PCAS (*P* < 0.05). The C-index of the nomograms was 0.706 (95% CI 0.654–0.758), and the calibration curves showed good agreement between the nomogram prediction and actual observation.

**Conclusion:**

PCAS is a rare cancer that is prone to have poor prognoses. To understand PCAS more thoroughly, more cases with adequate information are needed.

## Introduction

Angiosarcoma originates from vascular endothelial cells and occurs in the skin. Angiosarcoma is most commonly found in the head and neck, followed by soft tissue and mammary glands, liver, spleen and bone [1–3]. Primary cardiac angiosarcoma (PCAS) cases are extraordinarily rare but comprise most of the malignant cardiac tumour cases. PCAS originates from vascular endothelial cells or vascular endothelial cells that differentiated from mesenchyme cells [4, 5].

Much of the literature emphasizes that a majority of PCAS cases occur in the right atrium [6–9]. Angiosarcoma mainly occurs in the right atrium but can also occur in the right ventricle [10].The most important feature of cardiac angiosarcoma is invasive growth and metastases, which often infiltrate the myocardium, valves, pericardium, and even the coronary artery. Invasive growth is the main cause of high malignancy and poor prognoses for angiosarcoma [11]. Early diagnosis of angiosarcoma is still very difficult, and the clinical manifestations of angiosarcoma are atypical and can manifest as chest tightness, shortness of breath, arrhythmia, or symptoms of distal metastasis [12]. Early research studies for cardiac malignancies showed that primary cardiac tumours occurred at a frequency of 0.0001–0.030% in adult autopsy series, of which 25% are malignant [13, 14]. Cardiac malignancies, despite accounting for a low proportion of systemic tumours, often have serious consequences because of the specificity of their location. Furthermore, patients are diagnosed when their chests are probed or at autopsy due to a lack of specific clinical manifestations. With the development of imaging techniques, such as ultrasound, CT (computed tomography), and magnetic resonance imaging, an increasing number of cases can be diagnosed at an early stage [15, 16].

Data from several studies suggest that males are usually affected more often than females [17]. The tumours are prone to local and distant metastasis. PET–CT (positron emission tomography–computed tomography) showed that the most common sites of metastasis were the lungs, bone, liver and brain, and the prognoses of these patients remain extremely poor [18, 19]. While surgery is usually the preferred method of treatment, the role of combination treatment with chemotherapy and radiation has not been well defined [20].

There have been little published data analysing the presentation, management and outcome of patients with PCAS. These studies are seriously limited by small patient numbers. Thus, this population-based study aimed to investigate the clinicopathological characteristics of PCAS and identify factors related to the prognosis of PCAS patients.

## Methods

This study was designed as a retrospective analysis. We analysed a total of 168 PCAS patients selected from the National Cancer Institute’s SEER database. The SEER programme, supported by the National Cancer Institute, collects information on cancer morbidity and survival rates from 18 population cancer registries in America.

The criteria for selecting the subjects were as follows: (1) subjects who had tumours with malignant behaviour located in the heart (ICD-O-3/WHO 2008 site code C38.0); (2) subjects who had tumours with squamous histology (ICD-O-3/WHO 2008 morphology code 9120); and (3) subjects who were diagnosed with the tumour between January 1, 1973 and December 31, 2013. The distributions of the categorical demographic and clinicopathological variables were analysed including sex ethnicity, histological grade, summary stage, tumour size, TNM stage, LN (lymph node) metastases, distant metastases, surgery and radiotherapy status.

### Statistical analysis

The demographic and clinicopathological variables were compared between age groups using the Chi square test and Fisher’s exact probability test. The Kaplan–Meier curve of DSS was estimated and compared via the log-rank test. The Cox proportional risk regression model was used for single-variable and multivariable analysis of DSS. Multivariate Cox regression analysis was used in single-variable analysis using variables that were significantly related to DSS. SPSS 21.0 (IBM Corporation) was used for statistical analysis. The statistical significance was determined as *P* < 0.05.

Nomogram development was based on the Cox regression model. An index of concordance (C-index) between the predicted probability and the observed outcome was calculated to evaluate the predictive performance. The predictive performance was also assessed using calibration plots to compare the nomogram prediction with the observed outcome. Construction and validation of the nomograms were conducted on statistical R software (3.3.1).

## Results

Demographic and clinicopathological characteristics of PCAS patients.

**Fig. 1:**
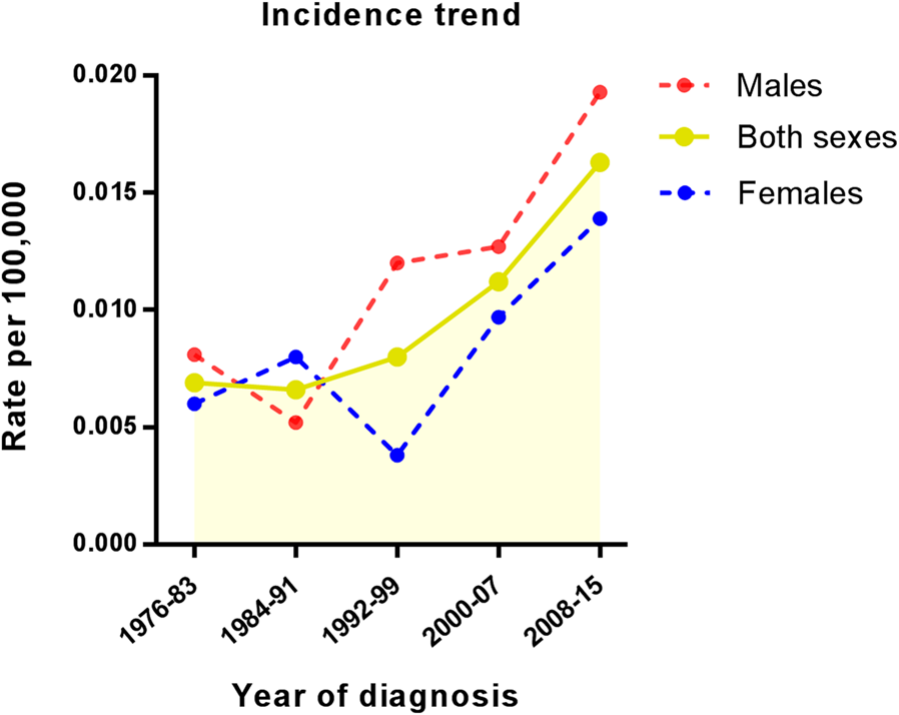
Analysis of incidence trend of primary cardiac angiosarcoma (incidence per 100,000) from 1976 to 2015

**Fig. 2:**
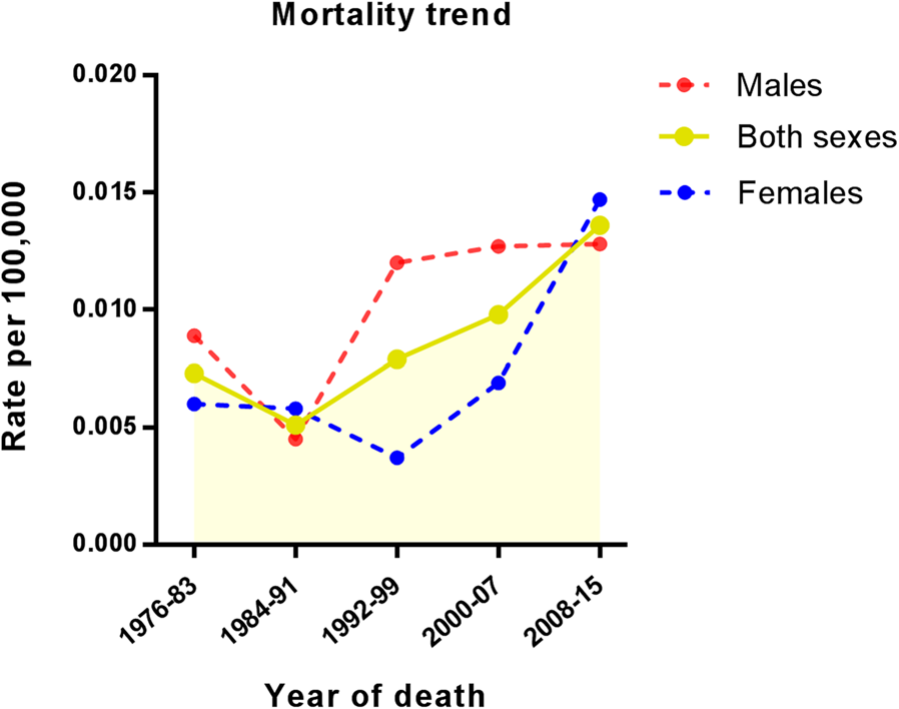
Analysis of mortality trend of primary cardiac angiosarcoma (incidence per 100,000) from 1976 to 2015

As shown in Figs. 1 and 2, there are clear trends of increasing age-standardized incidence and mortality rates per 100,000 person-years, which were computed for each sex, the overall factors and in 8-year calendar periods (1976–1983, 1984–1991, 1992–1999, 2000–2007, and 2006–2015).

Table 1 presents some of the main characteristics of PCAS patients. A total of 168 patients were included in this study: 74 (44.0%) patients were female and 94 (56.0%) were male. Of the initial cohort, the average age at diagnosis was 44.4 ± 15.5 years, and 91.9% were younger than 69, and most patients in our study were diagnosed with < 45 years old. Overall, most subjects (55.4%) were married. The race distribution was as follows: white (67.3%), black (19.0%), and other (13.7%). Among the 61 patients with records of pathological differentiation, 1 case was well differentiated, 5 were moderately differentiated, and the remaining cases were poorly differentiated (26) or undifferentiated (29). Tumour sizes, categorized as < 3 cm, 3–5 cm, and > 5 cm, were observed in 9 (5.4%), 32 (19.0%), and 63 (37.5%), respectively. Positive lymph node metastases and positive distant metastases accounted for the minority, considering missing data of half of the population. Localized stage indicated that an invasive neoplasm was confined entirely to the organ of origin. It may include intraluminal extension where specified, while regional stage meant the neoplasm that has extended (1) beyond the limits of the organ of origin directly into surrounding organs or tissues; (2) into regional lymph nodes through the lymphatic system; or (3) by a combination of extension and regional lymph nodes. A neoplasm that has spread to parts of the body remote from the primary tumour either by direct extension or by discontinuous metastasis (e.g. implantation or seeding) to distant organs, issues, or via the lymphatic system to distant lymph nodes is called distant stage. In summary, 44 (26.2%) cases were categorized as regional stage and 33 (19.6%) as localized stage, whereas 87 cases (51.8%) were categorized as distant stage. According to the year of diagnosis, we found that the incidence of PCAS continued to increase over time, and the majority of those PCAS occurrences were in younger subjects.

**Table 1:** Comparison of patient baseline characteristics between tumour-related death and alive or unrelated death

	Total	Alive or unrelated death	Tumour-related death	*P* value
Sex				
Female	74 (44.0%)	11 (42.3%)	63 (44.4%)	0.846
Male	94 (56.0%)	15 (57.7%)	79 (55.6%)	
Race				
White	113 (67.3%)	17 (65.4%)	96 (67.6%)	0.960
Black	32 (19.0%)	5 (19.2%)	27 (19.0%)	
Other	23 (13.7%)	4 (15.4%)	19 (13.4%)	
Age at diagnosis				
< 45	87 (51.8%)	18 (69.2%)	69 (48.6%)	0.053
≥ 45	81 (48.2%)	8 (30.8%)	73 (51.4%)	
Marital status				
Married	93 (55.4%)	14 (53.8%)	79 (55.6%)	0.426
Single	41 (24.4%)	8 (30.8%)	33 (23.2%)	
Other	27 (16.1%)	2 (7.7%)	25 (17.6%)	
Unknown	7 (4.2%)	2 (7.7%)	5 (3.5%)	
Histological grade				
I	1 (0.6%)	0 (0.0%)	1 (0.7%)	0.578
II	5 (3.0%)	2 (7.7%	3 (2.1%	
III	26 (15.5%)	3 (11.5%)	23 (16.2%)	
IV	29 (17.3%	5 (19.2%	24 (16.9%	
Unknown	107 (63.7%)	16 (61.5%)	91 (64.1%)	
AJCC TNM stage				
I	1 (0.6%)	11 (42.3%)	2 (1.4%)	0.064
II	9 (5.4%)	0 (0.0%)	8 (5.6%)	
III	10 (6.0%)	4 (15.4%)	6 (4.2%)	
IV	52 (31.0%)	0 (0.0%)	41 (28.9%)	
Unstaged	96 (57.1%)	11 (42.3%)	85 (59.9%)	
Tumour size				
< 3 cm	9 (5.4%)	3 (11.5%)	6 (4.2%)	0.104
3–5 cm	32 (19.0%)	6 (23.1%)	26 (18.3%)	
> 5 cm	63 (37.5%)	11 (42.3%)	52 (36.6%)	
Unknown	64 (38.1%)	6 (23.1%)	58 (40.8%)	
LN metastases				
Yes	14 (8.3%)	3 (11.5%)	11 (7.7%)	0.135
No	72 (42.9%)	15 (57.7%)	57 (40.1%)	
Unknown	82 (48.8%)	8 (30.8%)	74 (52.1%)	
Distant metastases				
Yes	49 (29.2%)	11 (42.3%)	38 (26.8%)	0.088
No	41 (24.4%)	8 (30.8%)	33 (23.2%)	
Unknown	78 (46.4%)	7 (26.9%)	71 (50.0%)	
Radiation				
Yes	44 (26.2%)	11 (42.3%)	33 (23.2%)	0.004
No/unknown	124 (73.8%)	15 (57.7%)	109 (76.8%)	
Surgery				
Yes	80 (47.6%)	14 (53.8%)	66 (46.5%)	0.489
No/unknown	88 (52.4%)	12 (46.2%)	76 (53.5%)	
Summary stage				
Localized	33 (19.6%)	6 (23.1%)	27 (19.0%)	0.248
Regional	44 (26.2%)	6 (23.1%)	38 (26.8%)	
Distant	87 (51.8%)	12 (46.2%)	75 (52.8%)	
Unstaged	4 (2.4%)	2 (7.7%)	2 (1.4%)	
Year of diagnosis				
1973–1988	21 (12.5%)	1 (3.8%)	20 (14.1%)	0.045
1989–2002	48 (28.6%)	4 (15.4%)	44 (31.0%)	
2003–2013	99 (58.9%)	21 (80.8%)	78 (54.9%)	
Sequence number				
1st	158 (94.0%)	25 (96.2%)	133 (93.7%)	0.622
2nd	10 (6.0%)	1 (3.8%)	9 (6.3%)	

A total of 142 patients died due to PCAS, while an additional 26 (14.7%) patients were still alive or died due to other causes. As noted in Table 1, most patients received radiation therapy (*P* < 0.05), and the year of diagnosis (*P* < 0.05) was significantly different between the two survival groups.

### Univariate and multivariable survival analysis

Among the patients in the study, 68 were valid follow-up patients with an average survival time of 27.6 months, and the remaining cases had missing data. Overall, the 1-year, 2-year, and 5-year DSS rates were 34.7%, 14.3%, and 10.2%, respectively. Variables potentially influencing DSS were further analysed using the Kaplan–Meier log-rank test. Univariate Cox analysis, as shown in Table 2, indicated that the age at diagnosis, radiotherapy, surgery, and tumour size was associated with DSS. However, the histological grade and metastases did not significantly affect the survival. Likewise, race, TNM stage, and summary stage were not statistically significant prognostic factors, as shown in Table 2.

**Table 2:** Univariate and multivariate analyses for DSS for patients identified in the SEER Program database from 1973 to 2013

Factors	Category	Univariate analysis			Multivariable analysis		
		HR	95% CI	*P* value	HR	95% CI	*P* value
Age	< 45/≥ 45	1.793	1.284–2.504	0.001	2.165	1.475–3.179	0.000
Race	White			0.936			0.162
	Black	0.982	0.640–1.506	0.934	1.478	0.904–2.414	0.119
	Other	0.913	0.558–1.494	0.717	0.807	0.469–1.389	0.439
Histological grade	Well			0.476			0.839
	Moderate	1.232	0.128–11.849	0.857	0.575	0.029–11.410	0.717
	Poor	2.604	0.347–19.527	0.352	1.889	0.209–17.053	0.571
	Undifferentiated	2.158	0.289–16.127	0.454	1.632	0.175–15.215	0.667
	Unknown	2.741	0.377–19.920	0.319	1.711	0.200–14.664	0.624
TNM stage	I			0.234			0.056
	II	0.584	0.123–2.771	0.498	0.386	0.026–5.760	0.490
	III	0.238	0.047–1.190	0.080	0.104	0.007–1.554	0.101
	IV	0.390	0.094–1.627	0.196	0.130	0.007–2.485	0.175
	Unknown	0.488	0.119–1.999	0.318	0.102	0.007–2.390	0.087
Tumour size	< 3 cm			0.001			0.026
	3–5 cm	1.673	0.635–4.406	0.297	1.955	0.698–5.475	0.202
	> 5 cm	2.049	0.812–5.170	0.129	3.182	1.149–8.809	0.026
	Unknown	3.572	1.425–8.953	0.007	3.878	1.387–10.841	0.010
LN metastases	No			0.126			0.730
	Yes	1.332	0.697–2.546	0.386	1.390	0.592–3.264	0.449
	Unknown	1.430	1.010–2.024	0.044	0.946	0.337–2.651	0.916
Distant metastases	No			0.157			0.372
	Yes	0.985	0.617–1.571	0.948	0.385	0.077–1.922	0.244
	Unknown	1.374	0.907–2.081	0.134	1.571	0.474–5.210	0.460
Radiation	Yes/no, unknown	1.751	1.053–2.911	0.031	1.629	1.049–2.529	0.030
Surgery	Yes/no, unknown	1.442	1.034–2.011	0.031	1.427	0.946–2.153	0.090
Summary stage	Localized			0.172			0.008
	Regional	0.691	0.44–1.086	0.109	0.771	0.422–1.410	0.399
	Distant	0.676	0.456–1.004	0.052	1.819	0.964–3.433	0.065
	Unstaged	0.656	0.161–2.678	0.557	0.294	0.063–1.363	0.118

Considering the limitations of univariate Cox analysis, the study uses multivariable Cox analysis to gain insights into the independent factors (Table 2). These findings are similar to the results of the univariate regression analysis. An age of ≥ 45 years (HR 2.165, CI 1. 475–3.179, *P* < 0.001), no radiotherapy (HR 1.629; 95% CI 1.049–2.529, *P* = 0.030), tumour size > 5 cm (HR 3.182; 95% CI 1.149–8.809, *P* = 0.026), and the summary stage (*P* = 0.008) were associated with worse PCAS-related survival. It is apparent from the data in Table 2 that patients who underwent radiotherapy had a better prognosis compared to those who did not undergo radiotherapy. Multivariable survival analysis also revealed that patients aged ≥ 45 years had a significantly worse DSS. Similarly, the tumour size and summary stage were associated with PCAS-related survival.

An exploratory subgroup analysis was conducted to identify at which age and stage the patients benefited from surgery or radiation. Compared to the < 45 age group, the patients at age ≥ 45 were significantly more benefit from radical therapy (HR 2.205; 95% CI 1.167–4.166, *P* = 0.015) and surgery (HR 2.212; 95% CI 1.084–4.516, *P* = 0.029). Moreover, patients at localized and regional stage had significantly improved survival after undergoing surgery (HR 2.063; 95% CI 1.071–3.973, *P* = 0.030) and radical therapy (HR 2.319; 95% CI 1.148–4.682, *P* = 0.019) than patients at distant stage (Tables 3, 4).

**Table 3:** Multivariate analyses for DSS when patients were stratified by age

Factor	Category	Multivariable analysis					
		Age < 45			Age ≥ 45		
		HR	95% CI	*P* value	HR	95% CI	*P* value
Race	White			0.963			0.006
	Black	1.029	0.479–2.213	0.941	3.141	1.419–6.953	0.005
	Other	1.139	0.452–2.870	0.783	0.635	0.283–1.427	0.272
Histological grade	I			0.952			
	II	0.000		0.838			0179
	III	1.602	0.116–22.162	0.725	0.158	0.014–1.742	0.132
	IV	1.478	0.095–22.982	0.780	0.076	0.007–0.892	0.040
	Unknown	1.165	0.094–14.448	0.906	0.182	0.019–1.704	0.135
Summary stage	Localized			0.105			0.024
	Regional	0.649	0.279–1.509	0.315	0.517	0.173–1.542	0.236
	Distant	1.795	0.796–4.048	0.158	1.435	0.467–4.412	0.529
	Unstaged	5.158	0.570–46.644	0.144	0.097	0.010–0.965	0.047
TNM stage	I						0.715
	II			0.372	9.084	0.368–224.343	0.177
	III	0.191	0.023–1.621	0.129	3.737	0.082–170.500	0.499
	IV	0.353	0.010–12.959	0.571	7.275	0.173–306.609	0.299
	Unknown	0.183	0.024–1.396	0.101	3.437	0.189–62.429	0.404
Tumour size	< 3 cm			0.199			0.179
	3 cm–5 cm	3.704	0.504–27.233	0.198	2.694	0.493–14.711	0.252
	> 5 cm	6.424	0.760–54.292	0.088	5.023	1.026–24.598	0.046
	Unknown	7.984	0.941–67.734	0.057	3.820	0.768–19.000	0.101
LN metastases	No			0.325			0.498
	Yes	4.595	0.619–34.081	0.136	0.550	0.162–1.871	0.339
	Unknown	0.000	0.000–2.237E+49	0.877	0.525	0.146–1.888	0.324
Distant metastases	No			0.720			0.237
	Yes	0.311	0.017–5.577	0.428	0.160	0.016–1.609	0.120
	Unknown	32,140.430	0.000–1.281E+58	0.869	1.779	0.372–8.510	0.471
Radiation	Yes/no, unknown	1.378	0.692–2.744	0.362	2.205	1.167–4.166	0.015
Surgery	Yes/no, unknown	0.947	0.490–1.831	0.429	2.212	1.084–4.516	0.029

**Table 4:** Multivariate analyses for DSS when patients were stratified by summary stage

Factor	Category	Multivariable analysis					
		Localized, regional			Distant		
		HR	95% CI	*P* value	HR	95% CI	*P* value
Age		1.724	0.969–3.068	0.064	2.765	1.572–4.866	0.000
Race	White			0.095			0.370
	Black	1.526	0.721–3.229	0.269	1.635	0.809–3.306	0.171
	Other	0.502	0.219–1.148	0.102	0.947	0.425–2.109	0.895
Histological grade	I			0.920			
	II	0.000	0.000–4.851E+56	0.888			0.343
	III	3.210	0.276–37.364	0.352	0.172	0.017–1.728	0.135
	IV	2.863	0.249–32.961	0.399	0.183	0.018–1.868	0.152
	Unknown	2.422	0.241–24.342	0.452	0.139	0.015–1.307	0.084
TNM stage	I			0.180			
	II	0.000	0.000–1.832E+56	0.877			
	III	0.000	0.000–3.701E+55	0.859			
	IV	0.000	0.000–8.405E+55	0.868			
	Unknown	0.000	0.000–4.593E+55	0.862	2.810	0.823–9.595	0.099
Tumour size	< 3 cm			0.112			0.102
	3 cm–5 cm	2.356	0.597–9.301	0.221	0.677	0.110–4.182	0.675
	> 5 cm	5.128	1.252–21.004	0.023	1.180	0.231–6.035	0.843
	Unknown	4.278	1.060–17.265	0.041	1.998	0.345–11.588	0.440
LN metastases	No						0.898
	Yes				1.224	0.435–3.444	0.702
	Unknown	0.160	0.019–1.318	0.088	1.223	0.377–3.967	0.738
Distant metastases	No						
	Yes						
	Unknown	11.736	1.292–106.592	0.029			
Radiation	Yes/no, unknown	2.319	1.148–4.682	0.019	1.432	0.790–2.595	0.237
Surgery	Yes/no, unknown	2.063	1.071–3.973	0.030	1.203	0.667–2.172	0.539

**Fig. 3:**
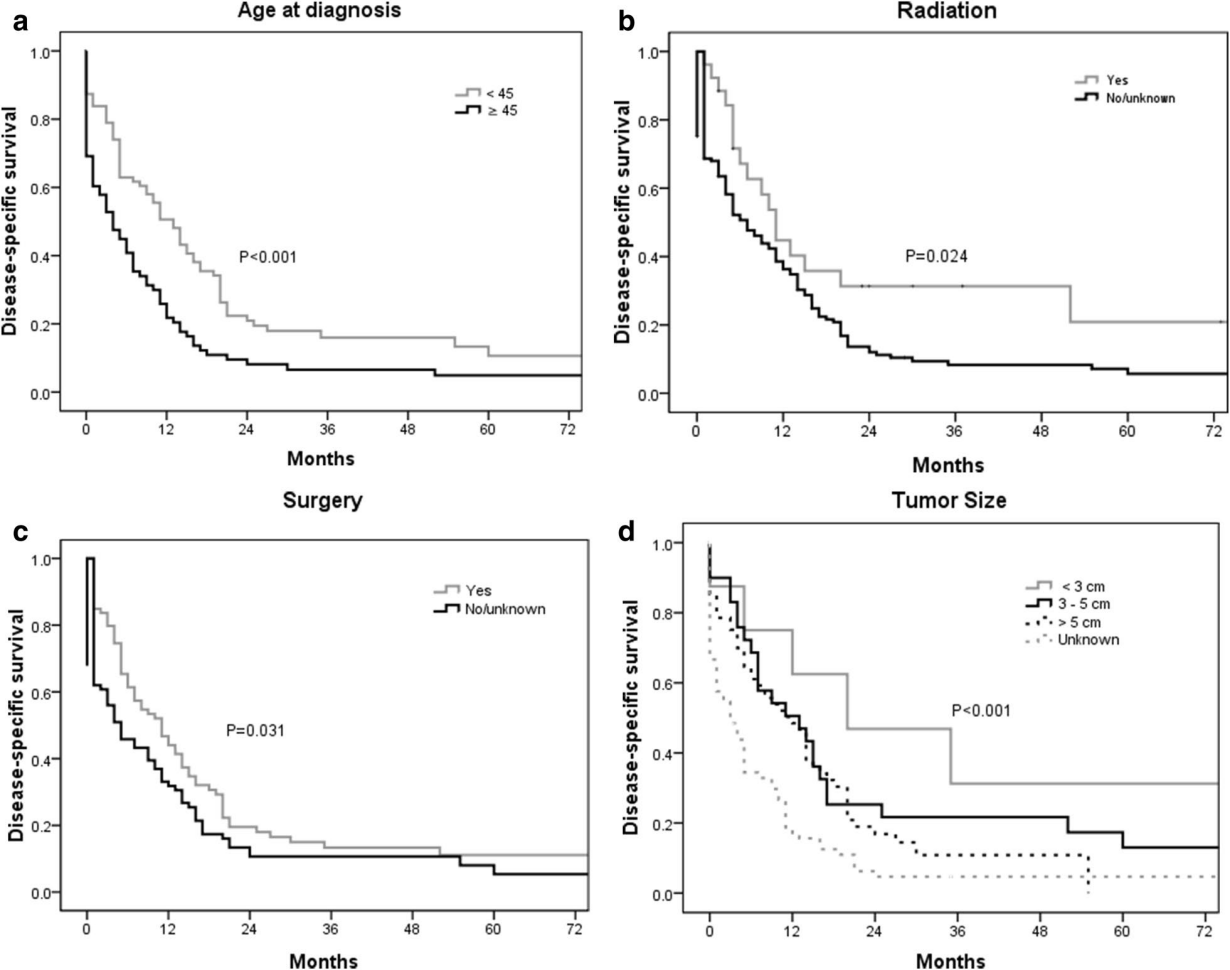
Kaplan–Meier estimated DSS in patients with primary cardiac angiosarcoma stratified by age (**a**), radiation (**b**), surgery (**c**), tumour size (**d**). *DSS* disease-specific survival

The DSS rate at 1, 2, 3, 4, 5 and 10 years was 34.7%, 14.3%, 13.0%, 11.7%, 10.2% and 8.8%, respectively. The median DSS was 7.2 years. As shown in Fig. 3a, the Kaplan–Meier analysis illustrates actuarial DSS according to the different factors. Survival was lowest for patients aged ≥ 45 years than for patients in another age group (*P* < 0.001). Figure 3b displays the Kaplan–Meier actuarial DSS if the patient received radiation. The survival of patients who had radiotherapy was significantly higher than that for those patients who did not have radiotherapy (*P* = 0.024). The DSS was significantly better for patients who underwent surgery compared to those who did not undergo surgical procedures (*P* = 0.031, Fig. 3c). Additionally, there were significant differences in DSS based on the tumour size (*P* < 0.001, Fig. 3d).

**Fig. 4:**
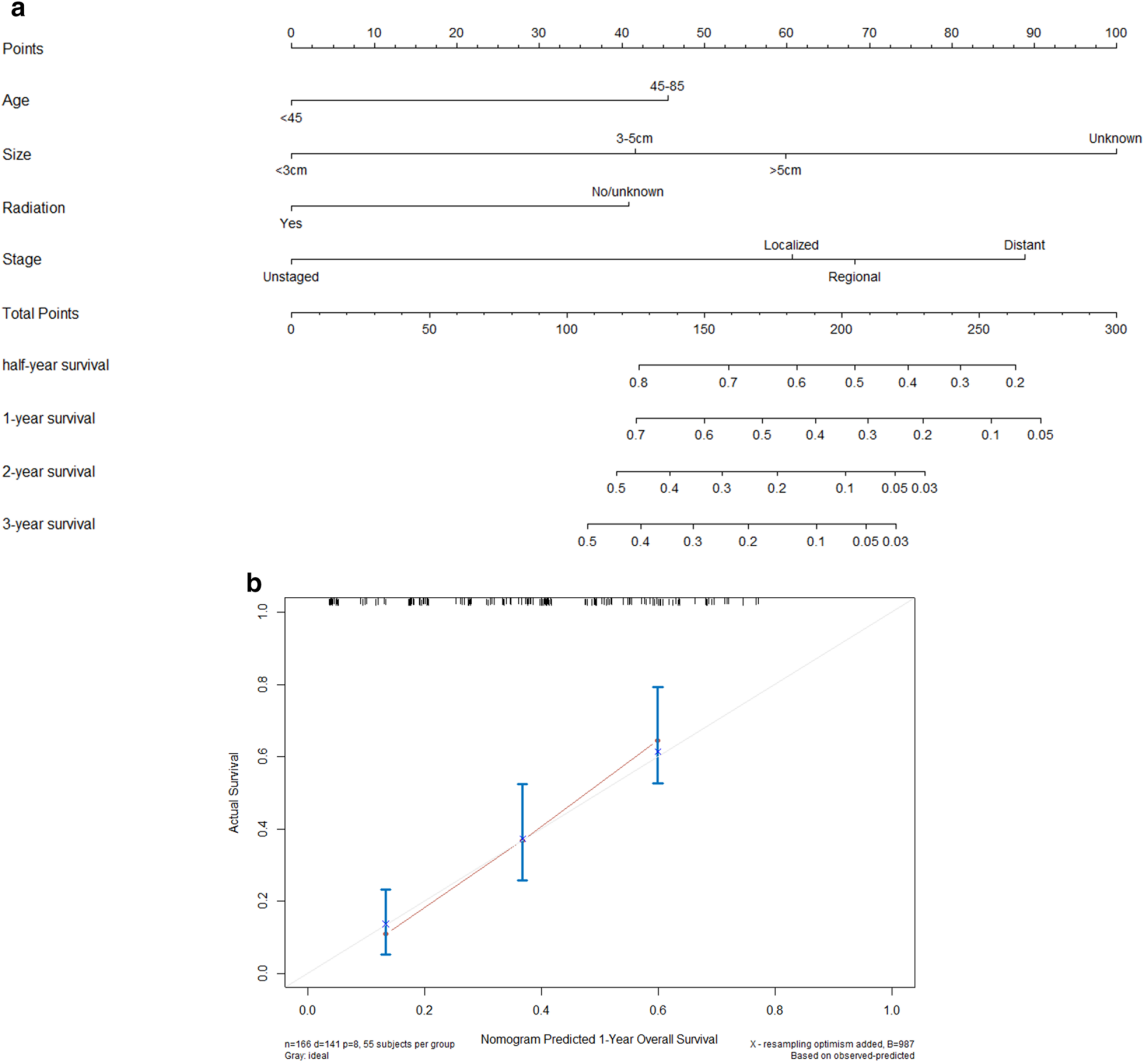
**a** Nomograms predicting 0.5-, 1-, 2- and 3-year DSS (**b**) of patients with primary cardiac angiosarcoma. **b** Calibration plots of the nomogram for 1-year DSS prediction. *X*-axis represents the nomogram-predicted probability of survival; *Y*-axis represents the actual OS probability. The blue circle overlaps the light blue line indicating near perfect calibration. Dots with bars represent nomogram-predicted probabilities along with 95% confidence interval

Furthermore, to predict the DSS of patients with PCAS, pre-treatment nomograms were established by a multivariate Cox regression model based on all meaningful variables that were factors for DSS (Fig. 4a). In the DSS prognostic model, the C-index was estimated to be 0.706 (95% CI 0.654–0.758). The calibration curve for the nomograms revealed no deviations from the reference line. The calibrations seemed to be satisfactory (Fig. 4b).

## Discussion

Regardless of the fact that primary cardiac tumours are relatively uncommon malignant neoplasms, angiosarcoma is the most common primary malignant tumour of the heart. There are little published data on PCAS due to its rarity. Furthermore, researchers have not studied the clinicopathological features and long-term follow-up reports of this entity in considerable detail. In the present study, we aim to conduct a deeper retrospective analysis of the clinical features, treatment and prognostic factors of PCAS, which may provide clear evidence for the clinical diagnosis and treatment of PCAS patients. We described the clinical characteristics of PCAS and identified variables influencing survival with the use of data in the SEER database between 1973 and 2013.

This study is based on the American population. We showed that the incidence of PCAS during the 40-year study period ranged from 0.0069/100,000 (1976–1983) to 0.0163/100,000 (2008–2015). Additionally, the mortality rate shows a similar trend as the incidence rate. Nonetheless, female patients had less incidence during 1990s, but then caught up with males. It is probably because the occurrence of tumours is not only related to genetic, genetics, lifestyle, and environmental deterioration, but also affected by personality and emotional stress. Therefore, the increase in morbidity may be related to the stress of women’s life in the recent years, as well as the use of contraceptives by women. The average age of PCAS patients is 44.4 years, and the ratio of male to female patients is 1.3:1. This male-dominant tendency is in agreement with the data analysis results of other research studies in Eastern and Western countries [10, 21]. In our study, there are some factors related with PCAS survival. According to our data, the tumour grade is not associated with survival. It may be because the grade information of 107 patients was unknown, which will affect the results.

A retrospective study by Hong investigated 18 cardiac AS patients [21]. At the time of diagnosis, 8 patients (44%) suffered from localized advanced diseases and 10 patients (56%) had metastatic disease. The median overall survival (OS) was 13 months for the entire cohort; the median OS was 19.5 months for those presenting with localized advanced AS and 6 months for those presenting with metastatic disease (*P* = 0.08). The median OS was improved (17 vs 5 months, *P* = 0.01) in patients who underwent primary tumour resection compared to patients with tumour in situ retention. Our study followed up 68 patients; we found that the median survival time of patients receiving surgery was improved (11 vs 7 months) compared with patients who did not undergo surgery. The DSS was significantly better for patients who underwent surgery compared to those who did not undergo surgical procedures; therefore, surgery should be considered as a good option for treatment in selected PCAS cases.

It is now well established from a variety of studies that cardiac AS is linked to poor prognosis. Resection of the primary tumour should be attempted when feasible, as the OS may be improved. Nevertheless, most patients die of disease progression. Fatima et al. analysed the data from 13 patients and concluded that primary angiosarcoma of the heart and great vessels is uncommon and associated with a grim prognosis [22]. Our data indicate the median DSS of patients who received radiotherapy significantly improved when compared with patients without radiotherapy (15 vs 6 months). As a result, it supports that surgery plus adjuvant therapy seems to be an option that improves DSS but it still needs further studies. Given that the majority of patients with this disease are relatively young, we recommend active multimodality treatment; however, the exact treatment plan must be individualized according to the characteristics of the patient.

PCAS has a poor prognosis with an average survival time of no more than 1 year [11, 23, 24]; however, the mean DSS of patients with PCAS in our cohort was 44 months, which was longer than the previous results and may be related to the certain treatment of some patients in this cohort. The treatment of primary cardiac tumours remains controversial, but surgery plays an essential role in the context of localized disease. The survival time for surgically treated patients ranged from 2 to 55 months and the median survival time was approximately 14 months [17], which is consistent with our study to an extent; the survival time of patients receiving surgery was ranging from 1 to 60 months, with a median survival time of 11 months.

Data from several sources have identified chemotherapy and/or radiotherapy as optimal options after radical or debulking surgery [25, 26]. In 1997, Kakizaki et al. published a paper in which they found that a PCAS patient survived for 30 months after surgery since he was treated with a combination of chemotherapy and immunotherapy [27]. Sinatra et al. published a paper in which they analysed data and concluded that the combination of surgical resection and radiation can reduce tumour size and eliminate symptoms, despite incomplete resections [28]. Much of the available literature on PCAS addresses the question of radiotherapy. Postoperative adjuvant radiotherapy can not only improve the local control rate but also decrease the recurrence rate after resectioning of the primary tumour [29, 30]. It is the first study that showed the same result in a bigger cohort which reinforces the importance of further studying these treatment options.

The nomograms have been used in the prognosis assessment of a variety of cancer patients and are considered to be superior to traditional staging assessments. From the nomograms graph, the approximate probability value of the dependent variable can be obtained from the value of the predictor variable. According to the specific situation of PCAS patients, the scores of the corresponding indicators are obtained. The total scores could be used to predict the survival rate, and individual prognosis can be evaluated. This project successfully constructed the nomograms prediction model based on independent risk factors determined by survival analysis, the C-index was estimated to be 0.706 (95% CI 0.654–0.758), which is helpful for the design of clinical treatment decisions and clinical research programmes.

There are certain unavoidable limitations in this study. First, the SEER database does not publish important clinical information such as chemotherapy regimen, surgical details, and complications. Therefore, some potential prognostic factors cannot appear in the Cox risk scale model. Another unavoidable limitation is the absence of a focused review and quality control of the pathology reports in this study.

## Conclusion

Through data analysis with the SEER database, we identified the clinicopathological characteristics, treatments, and outcomes of patients with primary cardiac AS. Our study provided evidence that the age and year of diagnosis may play an important role in the survival of PCAS patients. Clinically, radiotherapy and surgery can increase the long-term survival of patients with PCAS. We hope that this case series will provide the context for evaluating and treating PCAS.

## Data Availability

All data generated or analysed during this study are included in this published in this article. The datasets used and/or analysed during the current study are available from the corresponding author on reasonable request.

## Abbreviations

PCAS: primary cardiac angiosarcoma
SEER: Surveillance, Epidemiology and End Results
DSS: disease-specific survival
OS: overall survival
CT: computed tomography
PET–CT: positron emission tomography–computed tomography
LN: lymph node
